# Molecular Detection and Genotyping of *Enterocytozoon bieneusi* in Pigs in Shanxi Province, North China

**DOI:** 10.3389/fvets.2022.933691

**Published:** 2022-07-15

**Authors:** Zhen-Huan Zhang, Rui-Lin Qin, Ya-Ya Liu, Yang Zou, Jin-Jin Mei, Qing Liu, Wen-Wei Gao, Xing-Quan Zhu, Yu-Hong Ren, Shi-Chen Xie

**Affiliations:** ^1^College of Veterinary Medicine, Shanxi Agricultural University, Jinzhong, China; ^2^Heilongjiang Key Laboratory for Zoonosis, College of Veterinary Medicine, Northeast Agricultural University, Harbin, China; ^3^Research Center for Parasites & Vectors, College of Veterinary Medicine, Hunan Agricultural University, Changsha, China; ^4^Key Laboratory of Veterinary Public Health of Higher Education of Yunnan, College of Veterinary Medicine, Yunnan Agricultural University, Kunming, China

**Keywords:** *Enterocytozoon bieneusi*, pigs, prevalence, genotypes, Shanxi province

## Abstract

*Enterocytozoon bieneusi* is a common opportunistic intestinal pathogen that can cause acute diarrhea in immunosuppressed humans and animals. Though *E. bieneusi* has been widely detected in pigs around the world, little is known of its prevalence and genotype distribution in pigs in Shanxi province, north China. In this study, a total of 362 fecal samples were collected from pigs in three representative counties in north, south, and central Shanxi province, China. The prevalence and genotypes of *E. bieneusi* were investigated by nested PCR amplification of the ribosomal internal transcribed spacer (ITS) region of the ribosomal RNA (rRNA) gene. Overall, the prevalence of *E. bieneusi* in pigs in Shanxi province was 54.70% (198/362). Statistical analysis showed the difference in prevalence was statistically significant between regions (χ^2^ = 41.94, df = 2, *P* < 0.001) and ages (χ^2^ = 80.37, df = 1, *P* < 0.001). In addition, 16 genotypes of *E. bieneusi* were identified in this study by sequence analysis of the ITS region, including 15 known genotypes (EbpC, EbpA, EbpB, pigEb4, PigEBITS5, I, Henan-I, G, WildBoar 7, SH10, EbpD, CHC5, PigSpEb1, PigSpEb2, and CHG19) and one novel genotype (designated as PigSX-1). Phylogenetic analysis revealed that 14 known genotypes and the novel genotype were clustered into Group 1, whereas genotype I belonged to Group 2. To the best of our knowledge, this is the first report on the prevalence and genotypes of *E. bieneusi* in pigs in Shanxi province. These findings enrich the genetic diversity of *E. bieneusi* and provide the baseline data for the prevention and control of *E. bieneusi* in pigs in the study regions.

## Introduction

The phylum Microsporidia contains a large group of single-celled, obligate intracellular spore-forming parasites (more than 220 genera and 1,700 species). Of which, *Enterocytozoon bieneusi* is the most frequently detected species in humans (1). Although *E. bieneusi* infection in immunocompetent individuals is usually asymptomatic (2), acute diarrhea can occur in immunocompromised individuals, such as patients with AIDS (3). In addition, *E. bieneusi* has also been detected in a variety of mammals and birds (4). Humans and animals can be infected by *E. bieneusi* through contact with infected hosts or by ingesting spore-contaminated water or food (5).

Genotyping of *E. bieneusi* is based on amplification and sequencing of the ribosomal internal transcribed spacer (ITS) region of the rRNA gene, which has high single nucleotide polymorphisms (SNPs) (6). At present, over 500 genotypes of *E. bieneusi* have been identified, which are divided into 11 phylogenetic groups (5). Group 1 is the largest human-pathogenic group containing more than 300 genotypes (5). The prevalence of *E. bieneusi* in pigs varied, ranging from 10 to 93.70% worldwide (5). A number of genotypes within Group 1 identified in humans have also been found in pigs, suggesting that pigs could serve as a potential reservoir for *E. bieneusi* transmission to humans (5, 7, 8).

According to data from the National Bureau of Statistics of China, approximately 8 million pigs were produced and consumed in Shanxi province annually (http://www.stats.gov.cn/tjsj/ndsj/2019/indexeh.htm). However, little is known about the epidemiology of *E. bieneusi* in pigs in Shanxi province. In this study, the prevalence and genotypes of *E. bieneusi* in pigs in Shanxi province were investigated by using nested PCR amplification of the ribosomal ITS region. Meanwhile, phylogenetic analysis was conducted to evaluate the zoonotic potential of the *E. bieneusi* isolates.

## Materials and Methods

### Collection of Samples

In November 2020, with the permission of the farm owners, a total of 362 fresh fecal samples were randomly collected from pigs in three farms each in Shanyin county (39°52′ N, 112°81′ E) located in northern Shanxi province, Qi county (37°35′ N, 112°33′ E) located in central Shanxi province, and Jishan county (35°59′ N, 110°97′ E) located in southern Shanxi province. Approximately, 5–15% of samples were collected from each farm. All fecal samples were transported to the laboratory in a styrofoam box with ice packs immediately and stored at −20°C until genomic DNA extraction.

### DNA Extraction and PCR Amplification

The genomic DNA was extracted from each fecal sample (approximately 200 mg) using the E.Z.N.A.^®^ Stool DNA Kit (Omega Bio-tek Inc., Norcross, GA, USA) and stored at −20°C until used for subsequent PCR amplification. A nested PCR was performed to amplify the ITS region by using *E. bieneusi*-specific primers described in a previous study (9). Briefly, the reaction mixture (25 μl) contained 2.5 μl of 10×PCR Buffer (Mg^2+^ free), 1.5 mM of MgCl_2_, 2 μl of dNTP mixture (2.5 mM each), 1.25 U of *Ex*-Taq polymerase (Takara, Dalian, China), 1 μM of each primer, 14.75 μl of ddH_2_O, and 2 μl of DNA template. The conditions and cycling parameters were as follows: initial denaturation at 94°C for 5 min, followed by 35 cycles at 94°C for 30 s, annealing at 55°C for 30 s, 72°C for 40 s, and a final extension at 72°C for 10 min. To ensure the reliability of the results, each PCR amplification included a negative control (reagent-grade water) and a positive control (DNA of the *E. bieneusi* BEB6 genotype from sheep). Then, secondary products were checked by using 2.5% agarose gel and visualized under UV light after staining in ethidium bromide.

### Sequencing and Phylogenetic Analysis

All PCR products were sent to Sangon Biotech Co. Ltd (Shanghai, China) for two-directional sequencing on an ABI PRISM DNA Analyzer (Applied Biosystems, Foster City, CA, USA) using relevant internal primers for PCR amplification. The obtained sequences were aligned with the relevant sequences available in the GenBank database using Basic Local Alignment Search Tool (BLAST) and Clustal X to determine the genotypes of *E. bieneusi*. All samples with novel genotypes were sequenced two times to ensure the reliability of the data. The novel genotype was denominated according to the nomenclature established by Santin and Fayer (6). The phylogenetic tree was constructed by MEGA 7 using the Neighbor-Joining (NJ) method and Kimura 2-parameter model with 1,000 bootstraps (9).

### Statistical Analysis

In this study, the software SPSS V26.0 (SPSS Inc., Chicago, IL, USA) was used to analyze the correlation between prevalence and risk factors of *E. bieneusi* in pigs by Chi-square (χ^2^) test. Odds ratios (ORs) and their 95% confidence intervals (95%CIs) were calculated to identify risk factors. There was a significant difference in prevalence when the *p*-value was <0.05.

## Results

### Prevalence of *E. bieneusi* in Pigs in Shanxi Province

In this study, 198 of 362 fecal samples were detected to be positive for *E. bieneusi*, and the prevalence of *E. bieneusi* in pigs in Shanxi province was 54.70% (Table 1). Statistical analysis showed that the prevalence of *E. bieneusi* in pigs aged <6 months was 71.73% (170/237), which was significantly higher than that in pigs aged more than 6 months (22.40%, 28/125) (χ^2^ = 80.37, df = 1, *P* < 0.001). The prevalence of *E. bieneusi* in Qi county (22.06%, 15/68) was significantly lower than that of Shanyin county (53.15%, 59/111) and Jishan county (67.76%, 124/183) (χ^2^ = 41.94, df = 2, *P* < 0.001), respectively.

**Table 1:** Factors associated with prevalence of *Enterocytozoon bieneusi* in pigs in Shanxi province, China.

**Factor**	**Category**	**No. tested**	**No. positive**	**Prevalence% (95%CI)**	**OR (95%CI)**	***P*-value**
Region	Jishan	183	124	67.76 (60.99–74.53)	7.43 (3.87–14.25)	<0.001
	Qi	68	15	22.06 (12.20–31.91)	1	
	Shanyin	111	59	53.15 (43.87–62.44)	4.01 (2.02–7.94)	
Age	0 < month ≤ 6	237	170	71.73 (66.00–77.46)	8.79 (5.30–14.59)	<0.001
	6 > month	125	28	22.40 (15.09–29.71)	1	
Total		362	198	54.70 (49.57–59.82)		

### Genotype Distribution of *E. bieneusi* in Pigs

A total of 16 genotypes were identified by ITS sequence analysis, including 15 known genotypes (EbpC, EbpA, EbpB, pigEb4, PigEBITS5, I, Henan-I, G, WildBoar7, SH10, EbpD, CHC5, PigSpEb1, CHG19, and PigSpEb2) and one novel genotype (named as PigSX-1) (Table 2). Of which, EbpA (5.05%, 10/198), EbpC (34.34%, 68/198), and PigSpEb2 (22.22%, 44/198) were the predominant genotype in Qi county, Jishan county, and Shanyin county, respectively. Notably, genotype PigSpEb2 was detected in Shanyin county (55.70%, 44/79) and Jishan county (44.30%, 35/79), but not in Qi county. A comparison between the two age groups showed that PigSpEb2 was mainly distributed in young pigs (<6 months) (94.94%, 75/79). Almost all the genotypes identified in Jishan county were EbpC (98.55%, 68/69), which was mainly detected in young pigs (91.30%, 63/69). In addition, the novel genotype pigSX-1 (2.53%, 5/198) was only detected in pigs in Jishan county. Sequence analysis revealed that the novel genotype pigSX-1 showed a 98.71% similarity to the genotype EbpB (AF076041), with five SNPs.

**Table 2:** Genotype distribution of *Enterocytozoon bieneusi* in pigs in Shanxi province, China.

**Factor**	**Category**	**No. tested**	**No. positive**	**Genotypes (*n*)**
Location	Jishan	183	124	EbpC (68), PigSpEb2 (35), PigSX-1 (5), Henan-I (3), pigEb4 (3), I (3), WildBoar7 (2), PigEBITS5 (2), EbpA (1), CHG19 (1), CHC5 (1)
	Qi	68	15	EbpA (10), EbpB (4), EbpD (1)
	Shanyin	111	59	PigSpEb2 (44), EbpA (8), G (2), SH10 (2), EbpC (1), PigEBITS5 (1), PigSpEb1 (1)
Age	0 < month < 6	237	170	PigSpEb2 (75), EbpC (63), EbpA (16), PigSX-1 (5), EbpB (4), Henan-I (1), CHG19 (1), CHC5 (1), G (1), PigEBITS5 (1), PigSpEb1 (1), SH10 (1)
	month > 6	125	28	EbpC (6), PigSpEb2 (4), EbpA (3), pigEb4 (3), I (3), WildBoar7 (2), PigEBITS5 (2), Henan-I (2), EbpD (1), G (1), SH10 (1)
Total		362	198	PigSpEb2 (79), EbpC (69), EbpA (19), PigSX-1 (5), EbpB (4), pigEb4 (3), PigEBITS5 (3), I (3), Henan-I (3), G (2), WildBoar7 (2), SH10 (2), EbpD (1), CHC5 (1), PigSpEb1 (1), CHG19 (1)

### Phylogenetic Relationship Based on ITS Locus

**Figure 1:**
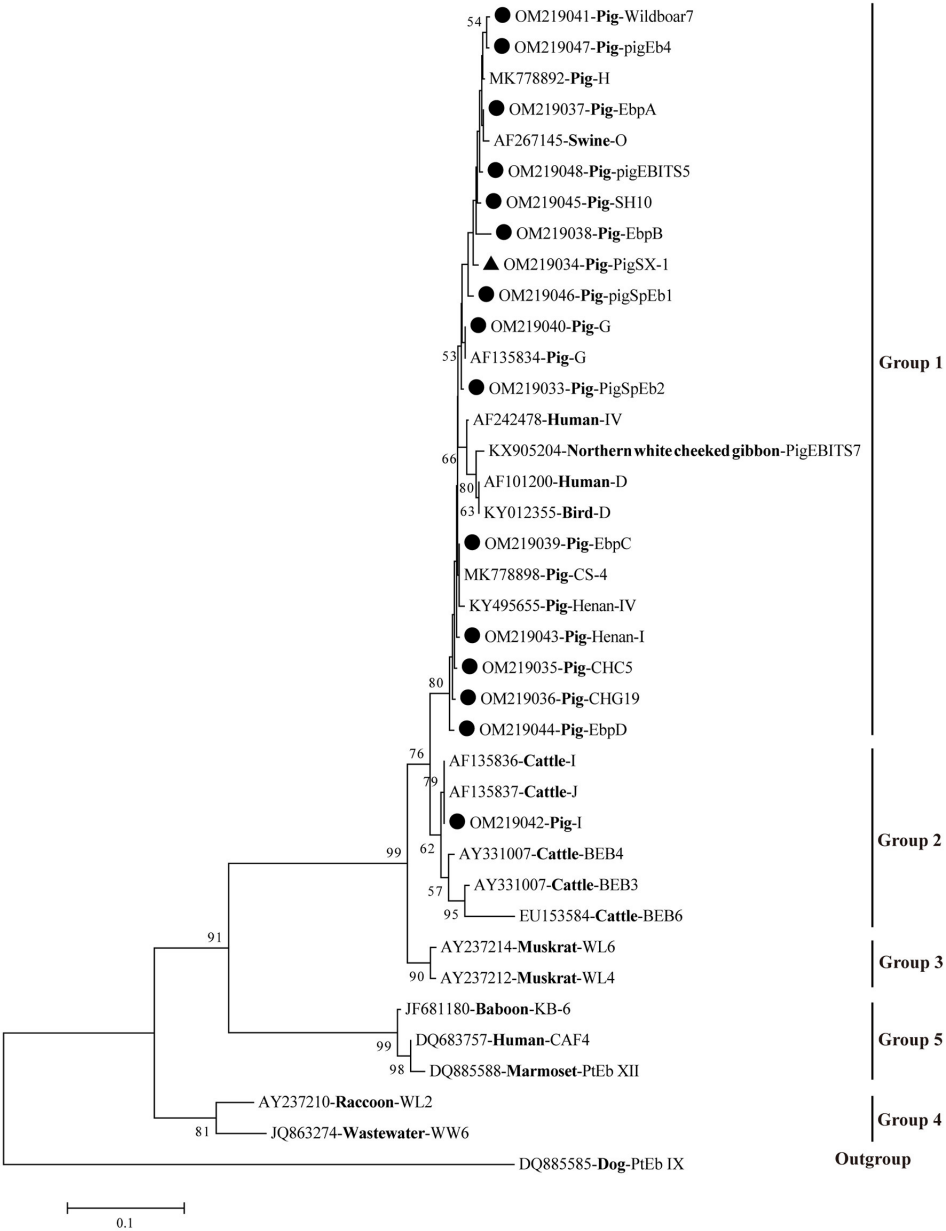
Phylogenetic relationships of *E. bieneusi* genotypes based on ITS sequences. Obtained ITS sequences in the present study were marked with black circles (• known genotypes) and black triangle (▴ novel genotype), respectively. The bootstrap value <50% was hide.

A phylogenetic tree was used to evaluate the genetic relationship of the 16 genotypes of *E. bieneusi* obtained in this study. The results showed that all 15 genotypes were clustered into Group 1, except for genotype I, which belonged to Group 2 (Figure 1).

### Nucleotide Sequence Accession Numbers

The 16 representative ITS sequences of *E. bieneusi* obtained in this study were deposited in the GenBank database under accession numbers OM219033-OM219048.

## Discussion

*E. bieneusi* has caused economic losses to the pig industry worldwide since it was detected in Sweden in 1996 for the first time (10). The results obtained in this study showed that the overall prevalence of *E. bieneusi* in pigs in Shanxi province was 54.70% (198/362), which was higher than that in pigs in most provinces in China (11–17) (Table 3), Thailand (14.75%, 36/244) (18), Japan (33.33%, 10/30) (19), Spain (22.58%, 42/186) (20), Massachusetts, USA (31.68%, 64/202) (21), and Malaysia (40.67%, 183/450) (22). However, the prevalence of *E. bieneusi* in pigs in Shanxi province was lower than that in two provinces in China (23, 24) (Table 3) and Brazil (59.34%, 54/91) (25). Regional differences in the prevalence of *E. bieneusi* may be related to geographical locations, sample volumes, breeding management, and ecological factors.

**Table 3:** *Enterocytozoon bieneusi* occurrence and genotypes identification in pigs in China.

**District**	**No. Positive/Total**	**Prevalence (%)**	**Genotypes**	**Year**	**References**
Beijing	108/257	42.02	**EbpC**, CAM5, wildboar12, CHS12, CM8, CTS3, Henan-IV, pigEBITS5	2020	(15)
Fujian	177/725	24.41	**EbpC**, EbpA, FJF, CHN-RR2, KIN-1, CHG7, CHS5, CM11, FJS, CHG23, G, PigEBITS, D	2019	(11)
Guangdong	19/72	26.39	**EbpC**, CHS5, GD1	2018	(14)
Hainan	88/188	46.81	**CS-4**, MJ14, CHG19, EbpA, HNP-I, HNP-II, HNP-III, HNP-IV	2020	(17)
Heilongjiang	267/563	47.42	**EbpC**, O, CS-4, EbpA, Henan-IV, PigEBITS5, EbpB, CC-1, CS-1, CS-3, CHN7, CS-10	2016	(12)
Henan	186/246	75.61	**EbpC**, EbpA, CHC5, CHG19, H, Henan-III	2019	(24)
Shaanxi province	442/560	78.93	**SZZD1**, SLTC2, SYLA5, CHG19, CHC5, SLTC3, SZZA2, EbpA, PigEBITS5, SHZA1, SZZC1, H, PigEB4, SYLC1, Henan-IV, SLTC1, SYLA1, SYLA2, CHS5, D, CHN7, CM6, SMXB1, SMXC1, SZZB1, SZZA1, SYLA3, SMXD1, SYLA4, SYLD1, CHG3, SZZD2, SHZC1, SMXD2	2018	(23)
Tibet autonomous Region	41/345	11.88	**EbpC**, CHS12, EbpD, PigEBITS5, GB11, GB31	2019	(13)
Xinjiang Uygur Autonomous region	389/801	48.56	**EbpC**, CHC5, CS-1, CS-4 CS-7, CS-9, D, EbpA, EbpD, H, PigEb4, PigEBITS5, WildBoar8, XJP-II, XJP-III	2019	(16)
Yunnan	58/200	29.00	**EbpC**, EbpA, YN1, Henan-IV, YN3, G, H, PigEBITS5, YN2, D	2018	(14)
Zhejiang	48/124	38.71	**EbpC**, EbpA, ZJ1, ZJ2, KIN-1, PigEBITS5	2018	(14)

*Prevalent genotypes of E. bieneusi in that study are shown in bold*.

There were significant differences in *E. bieneusi* prevalence between the two age groups, which was consistent with the results of a previous study (14). Some researchers argue that the probable reason for the higher prevalence of *E. bieneusi* in young pigs (<6 months) might be due to their imperfect immune system (23). However, a high prevalence of *E. bieneusi* was also found in older pigs in different areas of China (12, 14). The difference in *E. bieneusi* prevalence among these age groups indicated that geoecology, rearing conditions, and stocking density may be partially responsible for the variations in prevalence.

In this study, 15 known genotypes (PigSpEb2, EbpC, EbpA, EbpB, pigEb4, PigEBITS5, I, Henan-I, G, WildBoar7, SH10, EbpD, CHC5, PigSpEb1, and CHG19) and a novel genotype (PigSX-1) were identified in pigs in Shanxi province. Of which, genotype PigSpEb2 (39.90%, 79/198) was the predominant genotype, followed by EbpC (34.85%, 69/198) (synonyms: E, WL13, WL17, and Peru4) and EbpA (9.60%, 19/198) (synonym: F). This finding was not consistent with the results of previous studies, in which EbpC was detected as the predominate genotype in pigs in Zhejiang province, Guangdong province, Jilin province, and Tibet Autonomous Region in China (13, 14, 26). So far, the reasons for the difference in predominate genotypes of *E. bieneusi* in pigs from different study regions are still unknown. We reasoned that the geographical locations, pig breeds, and hygiene conditions might be responsible for the variations in predominate genotypes. Hence, more samples from diverse hosts in the study areas should be examined in the future to further clarify the possible patterns of prevalent genotypes of *E. bieneusi*.

Of those 16 identified genotypes, seven known genotypes (EbpC, EbpA, EbpB, PigEBITS5, I, EbpD, and CHG19) were commonly observed in humans (27), livestock (7, 28–30), non-human primates (NHPs) (31), wild animals (32), and water (33), posing a great threat to the public health. Particularly, genotypes EbpC and I were also found in squirrels and pet rabbits in China, respectively, which have close contact with humans (34, 35). Genotypes PigSpEb1 and PigSpEb2 were first identified in pigs in Spain in 2020 and 2021, respectively, but there was no data regarding the age patterns of the two genotypes in pigs (20, 36). Although our results revealed that younger pigs (<6 months) were more susceptible to PigSpEb1 and PigSpEb2, more investigations are still needed to confirm this in the future. A few studies have reported the presence of PigEb4, Henan-I, CHC5, Wildboar7, and SH10 in pigs, and further studies are warranted to clarify the host specificity and public health implications of these genotypes (24, 30, 37, 38). Phylogenetic analysis showed that 15 known genotypes were clustered into Group 1 and Group 2 (Figure 1). The novel genotype (PigSX-1) was clustered into Group 1, and was genetically closely related to zoonotic genotype EbpB, suggesting its importance and zoonotic potential.

## Conclusion

This study reported, for the first time, the prevalence of *E. bieneusi* (54.70%) in pigs in Shanxi province, north China, and a higher prevalence was observed in young pigs. Fifteen known *E. bieneusi* genotypes and one novel genotype (PigSX-1) were identified. Fifteen genotypes were clustered into Group 1, suggesting that these infections may not only be a veterinary issue but also a public health concern. These findings enriched the global genetic diversity of *E. bieneusi* and provided baseline data for the prevention and control of *E. bieneusi* infection in pigs in the study regions.

## Data Availability Statement

The original contributions presented in the study are included in the article/supplementary material, further inquiries can be directed to the corresponding authors.

## Ethics Statement

Ethical review and approval was not required for the animal study because this is not applicable. Written informed consent was obtained from the owners for the participation of their animals in this study.

## Author Contributions

S-CX, X-QZ, and Y-HR conceived and designed the experiments. Z-HZ performed the experiments, analyzed the data, and wrote the paper. J-JM, R-LQ, and Y-YL participated in the collection of fecal samples. W-WG and Y-HR participated in the implementation of the study. S-CX, QL, YZ, and X-QZ critically revised the manuscript. All authors have read and approved the final version of the manuscript.

## Funding

Project support was provided by Fund for Shanxi 1331 Project (Grant No. 20211331-13), the Special Research Fund of Shanxi Agricultural University for High-level Talents (Grant No. 2021XG001), the Yunnan Expert Workstation (Grant No. 202005AF150041), and the Veterinary Public Health Innovation Team of Yunnan Province (Grant No. 202105AE160014).

## Conflict of Interest

The authors declare that the research was conducted in the absence of any commercial or financial relationships that could be construed as a potential conflict of interest.

## Publisher's Note

All claims expressed in this article are solely those of the authors and do not necessarily represent those of their affiliated organizations, or those of the publisher, the editors and the reviewers. Any product that may be evaluated in this article, or claim that may be made by its manufacturer, is not guaranteed or endorsed by the publisher.
